# Sensitivity and specificity of neuropathy diabetes score, neuropathy symptoms score, diabetic neuropathy score and esthesiometry compared with the gold standards Michigan neuropathy screening instrument (MNSI) and Beck depression inventory (BDI)

**DOI:** 10.1186/1758-5996-7-S1-A199

**Published:** 2015-11-11

**Authors:** Lisiane Stefani Dias, Otto Henrique Nienov, Maria Cândida Ribeiro Parisi, Helena Schmid

**Affiliations:** 1 Universidade Federal do Rio Grande Do Sul, Porto Alegre, Brazil

## Background

In a previous study, we observed that the Results of an organized questionnaire to assess the presence of diabetic neuropathy (Diabetic Neuropathy Symptoms-DNS) were associated with the presence of scores of depressive symptoms (BDI ≥10).

## Objective

To evaluate how different scores for the presence of symptoms/signs of neuropathy (Neuropathy Diabetes Score-NDS; Neuropathy Symptoms Score-NSS; DNS and esthesiometry) had sensitivity and specificity, compared to the gold standard score of Michigan (MNSI) (≥2,5) and the gold standard score of Beck depressive symptoms (BDI).

## Materials and methods

207 patients with Diabetes type 2 were evaluated with MNSI, BDI, NDS, esthesiometry, NSS and DNS.

## Results

**Figure 1:**
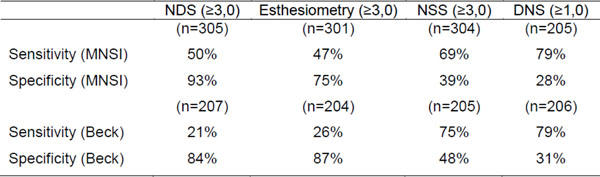
Sensitivity and specificty of NDS, esthesiometry NSS and DNS, compared to MNSI and BDI.

Questionnaires used to define the presence of polyneuropathy have a similar sensitivity for the detection of symptoms of depression (70 to 80%), while the physical examination for the presence of polyneuropathy (NDS) and esthesiometry has a sensitivity of ±50% and specificity of ±85% compared to MNSI and a sensitivity of ±23% and specificity of ±85% when compared to BDI (Figure 1). The symptom questionnaires have sensitivity and specificity of±75% and±35%, respectively, for both MNSI and BDI.

## Conclusions

We suggest not to use only questionnaires to define the presence of neuropathy in diabetic patients-in daily practice, physical examination (MNSI or NDS) must be used.

